# Biofertilizer Activity of *Azospirillum* sp. B510 on the Rice Productivity in Ghana

**DOI:** 10.3390/microorganisms9092000

**Published:** 2021-09-21

**Authors:** Elsie Sarkodee-Addo, Chihiro Tokiwa, Patrick Bonney, Daniel Asiamah Aboagye, Alex Yeboah, Samuel Oppong Abebrese, Ralph Bam, Eric Kwesi Nartey, Shin Okazaki, Michiko Yasuda

**Affiliations:** 1 Plant Microbiology Laboratory, Tokyo University of Agriculture and Technology, Saiwaicho 3-5-8, Fuchu 183-8509, Tokyo, Japan; elsieaddo67@yahoo.com (E.S.-A.); s218271t@st.go.tuat.ac.jp (C.T.); sokazaki@cc.tuat.ac.jp (S.O.); 2 West Africa Center for Crop Improvement, College of Basic and Applied Science, University of Ghana, Accra PMB 30, Ghana; gillbonney@gmail.com; 3 Department of Soil Science, School of Agriculture, University of Ghana, Accra P.O. Box LG 245, Ghana; daaboagye003@st.ug.edu.gh (D.A.A.); enartey@ug.edu.gh (E.K.N.); 4 CSIR-Savanna Agricultural Research Institute, Tamale P.O. Box TL 52, Ghana; lexisyeboah37@gmail.com (A.Y.); sam555oppa@yahoo.com (S.O.A.); 5 CSIR-Crops Research Institute, Kumasi P.O. Box 3785, Ghana; ralphbam@yahoo.com

**Keywords:** endophyte, plant growth promoting rhizobacteria, bio-fertilizer, microbial interaction, rice cultivars

## Abstract

Rice production in Ghana has become unsustainable due to the extremely nutrient-poor soils. It is caused by inadequate soil fertility management, including the inefficient application of fertilizers. A practical solution could be the biofertilizers, *Azospirillum* sp. B510. We performed field trials in Ghana and Japan to compare the effects of B510 colonization on selected Ghanaian rice varieties grown. The B510 inoculation significantly enhanced the rice cultivars’ growth and yield. The phenotypic characteristics observed in rice varieties Exbaika, Ex-Boako, AgraRice, and Amankwatia were mainly short length and high tillering capacity. These features are attributed to the host plant (*cv.* Nipponbare), from which the strain B510 was isolated. Furthermore, *Azospirillum* species has been identified as the dominant colonizing bacterium of rice rhizosphere across a diverse range of agroecologies in all major rice-growing regions in Ghana. Our results suggest that the utilization of B510 as a bio-fertilizer presents a promising way to improve rice growth, enhance soil fertility, and sustain rice productivity in Ghana.

## 1. Introduction

Rice (*Oryza sativa* L.) is a staple food source for over half the world’s population, with a volume of over 700 million tons [1,2]. It is an annual crop considered a perennial cereal with great economic importance [3]. Globally, the average rice consumption in 2018 was about 488 million tons, and Asia accounts for about 90% of production and consumption [4]. However, in Sub-Saharan Africa (SSA), rice consumption as food is increasing rapidly. In recent decades, rice demand among consumers has proliferated across Ghana due to the increased population, urbanization, and consumer-driven changes away from traditional food staples such as maize and cassava [5,6,7,8]. The demand rate has increased over the last two decades, and domestic rice production has not kept pace with this surge, fulfilling less than 40% of the national consumption [9,10]. This observed low yield has been attributed to several factors including nutrient-poor soils resulting from inadequate soil fertility management, recurrent droughts during the growing season, and the standard of the machinery used in production [9,11]. Consequently, farmers have been forced to rely on the application of chemical fertilizers and herbicides in order to maximize output and fulfill the increasing demand for rice. Extensive use of agrochemicals during crop production can lead to severe environmental problems, including residual soil, water, and food contamination, along with loss of microbial diversity. These factors compromise soil quality and thus pose a threat to human health and the environment [12].

The negative effects of the prolonged use of agrochemicals during crop production has driven research to find an innovative alternative approach in which beneficial microbes, such as plant growth-promoting rhizobacteria (PGPR), could be applied in the form of a biofertilizer [13,14,15,16]. PGPR, as soil-borne bacteria, enhances soil productivity to support plant growth [17]. The mechanisms of PGPR to promote plant productivity were reported to include biological nitrogen fixation, phosphorus solubilization, and the production of both siderophores and phytohormones [18,19]. These beneficial microorganisms belong to the phyla, Proteobacteria, Actinobacteria, and Firmicutes, with the most common genera being *Azospirillum, Azotobacter, Bacillus, Pseudomonas*, and *Rhizobium* [20,21,22]. It is well documented that PGPR enhances plant growth and improves ecosystem diversity by stimulating root growth [23] as well as increasing shoot biomass [24] and the uptake of water and nutrients by plants [25]. Many studies have demonstrated a mutualistic relationship between PGPR and crops, most notably in the Poaceae family, in which plant growth and grain yield are significantly increased by the association [26,27]. Numerous strains of the endophytic PGPR *Azospirillum* have been isolated from the roots and stems of these crops [28].

Members of the genus *Azospirillum* have been demonstrated to enhance plant health and crop productivity by several mechanisms, including metabolizing plant growth regulators [29], boosting plants immunity against pathogens [30], and by acting as biological nitrogen fixators [31]. The symbiotic association between *Azospirillum* and rice plants is prevalent. The symbiotic association between *Azospirillum* and rice plants is widespread in the paddy environment. Particularly during the vegetative phase, it is essential to growth promotion and largely contributes to enhancing crop productivity [32]. Beneficial interaction between microbes and crops profoundly impacts productivity and influences nutrient cycling, thereby altering soil fertility, leading to improved plant health. The availability of essential soil nutrients, such as nitrogen and phosphorus, is limited in tropical paddy fields, restricting plant health and crop productivity. However, previous studies have demonstrated a significant effect of PGPR on soil fertility under field conditions due to their pronounced influence on nutrient mineralization and organic matter decomposition and are not applied to replace fertilizers [20,33,34,35].

*Azospirillum* sp. B510, which was isolated from the stems of rice (*Oryza sativa cv*. Nipponbare) in Japan [36], has been formulated into a commercial product and applied as a biofertilizer in diverse crop production systems [37,38]. The application of *Azospirillum* sp. strain B510 during rice production demonstrated a significant influence on immunity against pathogens, plant growth, and yield output [39,40,41], which could be a potential candidate for enhancing and sustaining rice production in Ghana. However, with limited access to biofertilizer and the lack of farmers’ awareness of such farm inputs in Ghana, its utilization could be challenging during crop production.

In the face of inefficient utilization of fertilizers, combined with their harmful effects when used long-term, it is critical for crop production in Ghana that a method is found to utilize naturally available resources such as PGPR. In this study, we investigated the growth-promoting effect of B510 on Ghanaian rice cultivars in Japan and we performed an inoculation test on rice productivity in Ghana. Considering the extremely nutrient-poor soil conditions of paddy fields in Ghana, the field experiment carried out in Japan evaluated the effects of both low nitrogen and standard nitrogen soil conditions. Multiple field experiments were established to examine the impact of inoculation with B510. Moreover, in this study, we examined the potential of B510 application to improve plant growth and productivity in selected Ghanaian rice cultivars.

## 2. Materials and Methods

### 2.1. Experimental Site Description and Field Management

The experimental field in Japan was conducted in the Experimental Farm of Field Science Center, Tokyo University of Agriculture and Technology located Fuchu-honmachi, Tokyo (35°41′ N, 139°29′ E, 46 AMSL). The soil of the paddy field is classified as alluvial soil [42]. Phosphorus (P_2_0_5_) and potassium (K_2_O) fertilizers were applied at 30 kg ha^−1^ as basal fertilizers. Ammonium sulfate (NH_4_SO_4_) was applied at 20 kg ha^−1^ as top-dressers at five different times (at two weeks intervals). Rice seeds were sterilized using 0.1% Sumition and 0.5% Sportac Stana SE (Sumitomo Chemical, Japan) for 24 h, then thoroughly washed immediately in tap water at five separate times for 15 min. Seeds were later allowed to imbibe in tap water for about 72 h (water is changed after every 24 h) till germination. Germinated seeds were sown in nursery boxes on 7 May 2020. Seedling at the fourth leaf stage was transplanted to the paddy field on 21 May. The planting area was 120 m^2^, with a 30 cm × 30 cm planting distance (Figure S1). The field was submerged throughout the experiments by irrigation.

The first field in Ghana was in the *Ohayo farms* located at Nsawam within the semideciduous rainforest ecological zone (06°8′48″ N, 00°53′58″ W, 170 m). This area receives bimodal rainfall (1300–1500 mm) annually, allowing for two cropping seasons. The soils covering the area are classified as Acrisol and characterized as forest ochrosols with a friable consistency in a fine granular structure (FAO/ISRIC/ISSS, 1998). Before transplanting, seeds were soaked for 24 h in clean water and incubated in a jute sack (25 kg) filled to half its capacity for 30–36 h for germination. Seeds were sown on prepared seedbed plots (close to the field) of 1 m × 10 m and raised 5 cm above the original field level. Seedlings at the third leaf stage were inoculated with the commercial formulation of B510 (30 mL/L) and transplanted to the paddy field two days later at a spacing 25 cm × 20 cm, and a planting density of 20 hills/m^2^. The experimental plots were laid out in a randomized complete block design with three replicates arranged in a 2 × 2 × 4 factorial scheme. Field management was by the local agronomic practices with a split fertilizer application rate of 30:30:30 kg ha^−1^ NPK and 30 kg ha^−1^ urea prior to the reproduction stage. The experiment was under an irrigation system. The data were collected in November 2019.

The second field was at the *Golinga* irrigation area within the Guinea Savanna agro-ecological zone (09°21′06.0″ N, 000°57′01.0″ W, 139 m), with unimodal rainfall (1100 mm), flash floods, and drought; all of which significantly impact crop production (Ghana Meteorological Agency, [43]). This site has one cropping season, and soils covering the area are classified as Ferric Acrisols/Ferric Lixisols [44]. Seeds were sown in prepared seedbeds on 8 August 2020. Seedlings at the fourth and fifth leaf stage were inoculated with the commercial formulation of B510 and transplanted to the paddy field three days later. In a split application, 60:60:60 kg ha^−1^ NPK was applied as a basal application to fertilized plots a week after transplanting, and a top-dressing of 30 kg ha^−1^ nitrogen was applied at the booting stage in the form of urea. Weeds were manually controlled (by hand-picking) whenever necessary, and this activity did not cause any effect on any experimental treatments. The field was submerged throughout the experiments by irrigation.

### 2.2. Rice Cultivars

We obtained certified rice seeds from three different sources during these experiments. For the field experiment in Japan, Ghanaian cultivars Ex-Amantin, Ex-Boako, Bawku-Market, and Zebila of the sub-species group *Indica* were obtained from the Genetic Resources Centre NARO (National Agriculture and Food Research Organization, Tsukuba, Ibaraki, Japan). We used Japonica rice (*Oryza sativa* cv. Nipponbare) as a control to confirm the positive PGPR effect of B510. At Nsawam, we used the commercial cultivar Exbaika, which is tolerant against rice blast disease. Golinga seeds of four commercial rice varieties AgraRice, Amankwatia, Exbaika, and Jasmine85, were obtained from the Rice Development Sector, CSIR: Savanna Agriculture Research Institute, Tamale, Ghana. Further characteristics of the rice cultivars used in this study are summarized in Table S1.

### 2.3. Azospirillium Inoculation of Rice Seedlings

Rice seedlings were inoculated with *Azospirillum* sp. B510, using the commercial bacterial solution Ine-Fighter^®^ (Mayekawa Co., Ltd., Tokyo, Japan). Seedlings were watered with 3.3 mL Ine-Fighter^®^ solution diluted in 1 L of sterilized distilled water per nursery box (approximately 2 × 10^7^ CFU/plant). Nursery grown seedlings were inoculated with B510 once, 2–10 days before transplanting, according to company’s instruction.

### 2.4. Colonization Assay Using Ds-Red-Tagged Azospirillum

Endophytic bacterial colonization and observation of lateral roots colonized by DsRed-tagged B510 was performed according to the procedures followed by Naher et al. [41]. Rice seeds were sterilized using 70% ethanol for 20 sec, followed by 5% sodium hypochlorite for 10 min and then thoroughly washed 5 times in sterilized distilled water. Sterilized seeds were soaked in Rice Growth media containing 1 mM KNO_3_ and the bacterial suspension was then dropped on the seeds (50 µL/seed, approximately 2 × 10^5^ CFU/plant). Plants were grown in a growth chamber (LPH-240SP; NK System, Osaka, Japan) under a regime of 16 h light:8 h dark at 25 °C for 14 days. The lateral root surface was observed using an Olympus IX71 fluorescence microscope (Olympus, Tokyo, Japan). The measurement of colony-forming units (CFU/g) inside plant tissues was weighted and surface sterilized using 70% ethanol for 15 s followed by 1% sodium hypochlorite for 30 s after which samples were thoroughly washed 3 times in sterilized distilled water. A sterilized mortar and pestle were used for crushing whole seedlings after adding 1 mL 0.85% sodium chloride; solutions were spread on nutrient broth media containing 50 mg L^−1^ polymyxin B and 50 mg L^−1^ streptomycin at appropriate dilutions. After incubation at 28 °C for 3 days, the number of antibiotic resistant colonies were counted.

### 2.5. Evaluation of Plant Growth and Yield

A range of agronomic characteristics was assessed at the vegetative and harvested stages to evaluate the impact of strain B510 on the growth performance of rice plants: plant height, tillers number, chlorophyll content (SPAD), root length, fresh and dry weight of both shoots and roots, and the number of panicles and grain yield (harvested stage only). Dry weight values were obtained by oven-drying at 60 °C for 72 h and weighted.

### 2.6. Statistical Analysis

Statistical analyses were performed using a one-way analysis of variance (ANOVA) followed by the Student-Newman-Keuls (SNK) test in R software [45]. Different lower-case letters represent significantly different values (*p* < 0.05).

## 3. Results

### 3.1. The Endophytic Colonization of Azospirillium sp. B510 in Japonica and Ghanaian Rice

**Figure 1:**
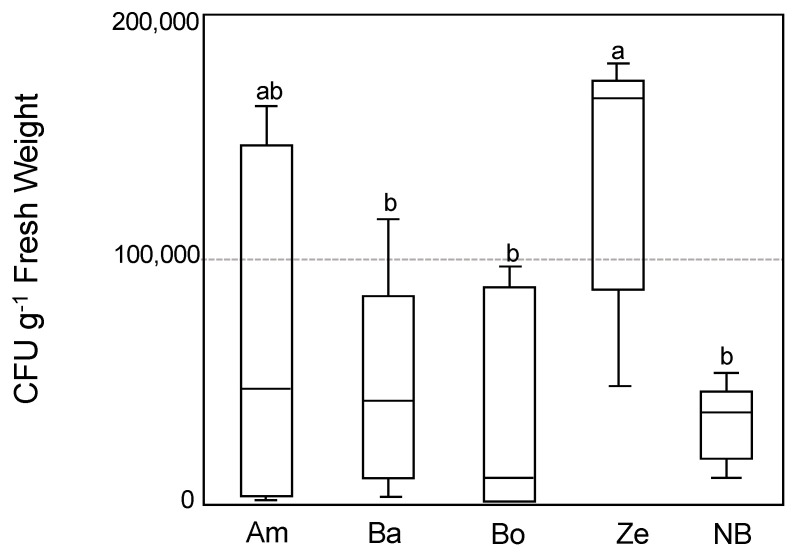
The endophytic colonization of *Azospirillum* sp. B510 on Japonica and Ghanaian rice. The endophytic colonization of DsRED-tagged B510 in the tissue of Ghanaian (Ex-Amantin (Am), Bawku-Market ***^a^*** (Ba), Ex-Boako (Bo), and Zebila (Ze)) and Japonica (Nipponbare (NB)) rice. Colonies were counted using dilution methods. Values presented are the average ± SD from 6 replicates of one plant each. Different letters indicate the significant differences between treatments (Student–Newman–Keuls (SNK) test, *p* < 0.05, *n* = 6).

In a previous study, some *Indica* rice cultivars showed a positive PGPR response to inoculating with B510 under low nitrogen conditions [33]. However, it was unclear whether this isolated strain from the *Japonica* rice cultivar could colonize *Indica* rice. Therefore, we visualized the colonization of B510 in four selected Ghanaian rice cultivars (Ex-Amantin, Ex-Boako, Bawku-Market, and Zebila) by performing an inoculation test using DsRED tagged strain and estimated the colonization effect under a field condition in Japan [46]. The result revealed a red fluorescence in each cultivar’s lateral roots (Figure S1), indicating that B510 had colonized the root surface. Besides, the display of colony-forming unit (CFU) by the endophytic colonization of DsRED tagged B510 inside whole plants shows that the greatest degree of B510-colonization was observed in the cultivars Zebila and Ex-Amantin, respectively (Figure 1). However, no significant difference was found between the CFUs of the Bawku-Market, Ex-Boako, and Nipponbare cultivars. These results indicated that B510 could colonize both the surfaces and inner tissues of Japonica and Ghanaian rice plants.

### 3.2. Effect of Azospirillium sp. B510 Inoculation on the Growth-Promotion in Ghana Rice in a Paddy Field in Japan

**Figure 2:**
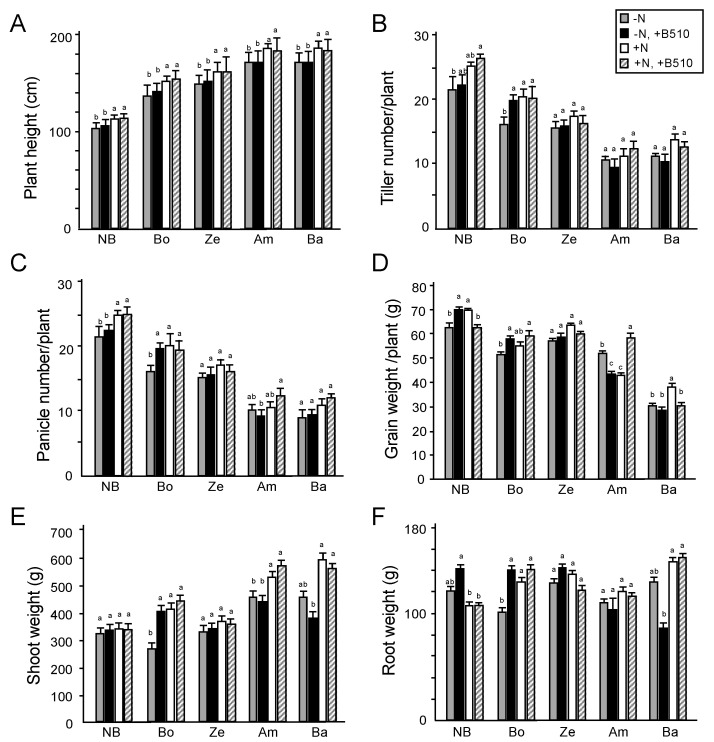
The effect of *Azospirillum* on plant growth under −N and +N conditions at 90 days after transplantation (harvesting stage). The plant growth characteristic assessed were plant height (**A**), tiller number (**B**), panicle number (**C**), grain weight (**D**), shoot weight (**E**) and root weight (**F**). The different rice cultivars used in this analysis were Nipponbare (NB), Ex-Boako (Bo), Zebila (Ze), Ex-Amantin (Am), and Bawku-Market (Ba). Statistical analysis was performed, the different letters (a, b, c) indicate the significant differences between treatments (Student–Newman–Keuls (SNK) test, *p* < 0.05, *n* = 6–9).

The effect of B510 inoculation on Indica rice cultivars has been previously demonstrated to be better under low-nitrogen than standard-nitrogen conditions [34]. To address whether plant growth was enhanced by B510 inoculation, we performed a field experiment in which we transplanted the seedlings of rice cultivars to paddies of either non-nitrogen (−N) or standard nitrogen (+N) application conditions and compared their agronomic traits (Figure 2). The Japonica rice cultivar Nipponbare was used as a positive control to assess the effect of the inoculation process by strain B510. The plant height increment observed among all the cultivars was uninfluenced by B510 inoculation but increased by +N conditions. The highest tiller number was recorded in the *cv.* Nipponbare (Figure 2B). Whilst tiller number among the cultivars was primarily influenced by nitrogen fertilization; we observed a positive influence of B510 inoculation in Ex-Boako; panicle numbers in the −N condition were greatest in Ex-Boako seedlings that had been inoculated with B510 (Figure 2C).

Conversely, B510 inoculated Ex-Amantin plants had significantly fewer panicles than the non-inoculated control plants (Figure 2C). Accordingly, grain weight was greater in the Nipponbare, Ex-Boako, and Zebila cultivars than −N and non-inoculated plants (Figure 2D). The grain weight of inoculated plants was lower than non-inoculated plants under the −N condition (Ex-Amantin) and both −N and +N conditions (Bawku-Market). The only evidence of a negative effect of B510 inoculation on shoot weight was observed in the Bawku-Market grown cultivars in the −N area (Figure 2E).

In Nipponbare, B510 inoculation significantly increased root weight under the +N condition, and fertilizer treatment reduced root growth (Figure 2F). Conversely, the root weight of Ghanaian rice cultivars was greater in plants treated with fertilizer (Figure 2F). The Ex-Boako plants inoculated were significantly different compared to control plants, which prove to be the most effective treatment, revealing an increase in root weight (Figure 2F). There was no significant difference between treatments of the Zebila and Ex-Amantin cultivars. Inoculated Bawku-Market showed an apparent significant decrease in root dry weight compared with plants treated with fertilizer (Figure 2F). These results suggest that Ex-Boako responded positively to B510 inoculation (which was exhibited in the grain yield) compared with the response observed among the cultivars Ex-Amantin and Bawku-Market.

### 3.3. Characteristic Response of Cultivars to Azospirillum sp. B510 Inoculation among Fertilization

**Figure 3:**
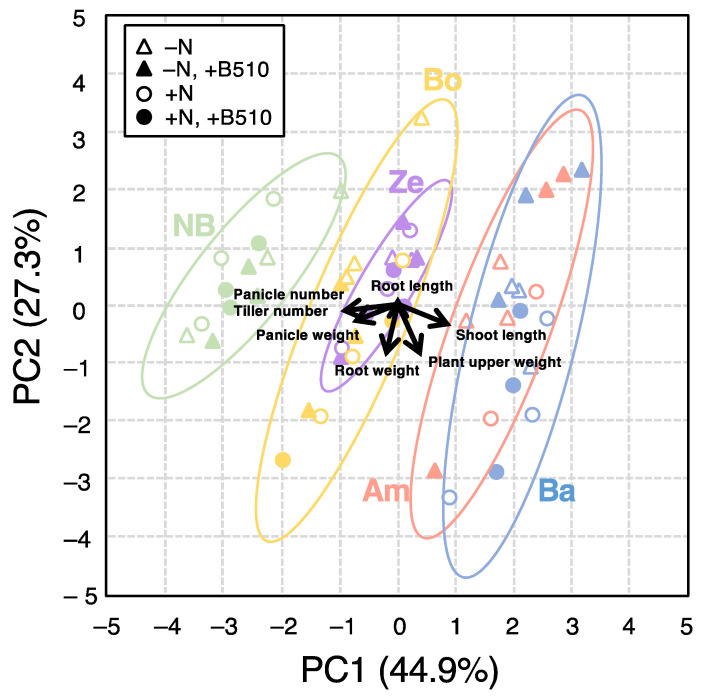
Principal component analysis (PCA) of rice cultivars with or without B510 inoculation. The plant growth characteristics (shoot length, plant upper weight, root length, root weight, tiller number, panicle number, and panicle weight) were assessed at the harvested stage. Rice cultivars are denoted by different colors as green-Nipponbare (NB), yellow-Ex-Boako (Bo), purple-Zebila (Ze), red-Ex-Amantin (Am), and blue- Bawku-Market (Ba). The length of labeled lines along an axis indicates the magnitude of correlation between original values of a trait and the principal component.

Principal component analysis (PCA) was performed to demonstrate how rice inoculation and fertilization application affected the rice cultivars’ growth-promoting traits and yield. Moreover, PCA was applied to compare the cultivar’s responses to the different treatments, particularly that of B510 inoculation. Statistical analysis showed that the growth-promoting characteristics and yield parameters associated with the rice cultivars responded differently among inoculated and fertilized samples. Two main components accounted for 74.7% of the variability observed in the data, with PC1 accounting for 48.8% of the total variation and PC2 accounting for 25.9% (Figure 3). Inoculation with B510 affected rice growth and yield regardless of nitrogen fertilization. PCA indicated positive correlations between plant height, root length and shoot dry weight but not for tiller number, panicle number, panicle weight, grain weight and root dry weight. Furthermore, PCA confirmed the negative impact of B510 inoculation on these parameters, with the exception of root length (Figure 3).

We examined the impact of inoculation on plant morphology, yield, and phenotypic characteristics. We observed that rice cultivars with short culms produced higher numbers of tillers, and conversely, the cultivars with long culms produced fewer tillers. Therefore, PC1 separated rice cultivars into two groups with clear distinction based on their culm size, regardless of fertilizer treatment. The cultivars with characteristically short culms were Nipponbare, Ex-Boako, and Zebila, whereas Ex-Amantin and Bawku-Market had long culms. Among the groups we observed, the cultivars Ex-Boako and Zebila overlapped in one group. Ex-Amantin and Zebila also coincided with the plot, suggesting similarity in genotype (thus *Indica*). The assessment of morphological traits also confirmed the positive effect of B510 inoculation on the growth of Nipponbare and Boako (as reflected by their higher grain weights) compared to the negative impact on Ex-Amantin and Bawku-Market (as reflected in their lower grain weights).

### 3.4. Effect of Azospirillum sp. B510 Inoculation on Growth and Yield of Commercial Rice Production in Ghana

We investigated the effect of B510 on the productivity of commercial rice cultivars grown in Ghana. Two separate field trials were conducted using different rice varieties and under different agro-ecology. Morphological and yield responses to B510 inoculation were evaluated at the vegetative and harvested stage.

**Figure 4:**
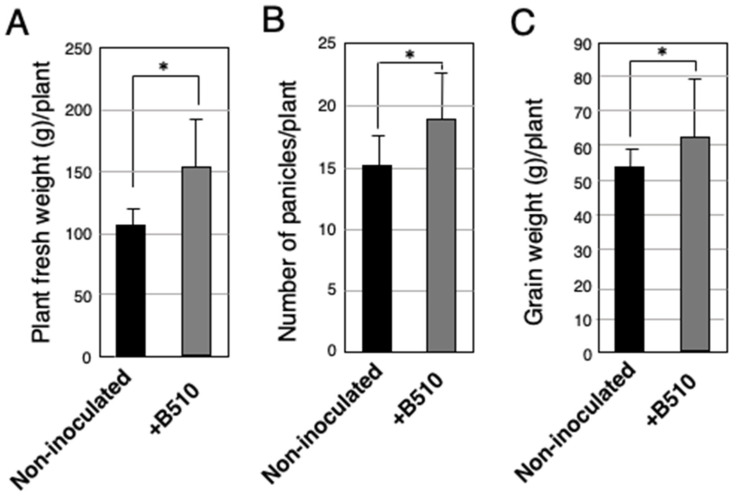
The effect of B510 inoculation on rice growth managed under field conditions in Nsawam, Ghana. A field test was performed in Ohayo-farm at Nsawam using cv. Ex-baika. Plants were grown in a nursery field and seedlings were inoculated with B510. After 2 dpi, the plants were transferred to the paddy field. Grain yield was measured after 2 months. Growth characteristics assessed were fresh weight of the upper grown parts (**A**), number of panicles (**B**), and grain weight per plant (**C**). Bar colors: non-inoculated control (black) and inoculated with B510 (gray). Values presented are the average ± SD from 10 plants each 7 plots. Statistical analysis was performed, the asterisk indicates the significant differences between treatments (Unpaired Student’s test, *p* < 0.05, *n* = 7).

In *Ohayo-farm*, we evaluated the effect of B510 inoculation on the most cultivated rice *cv.* Exbaika in a field trial managed under similar agronomic practices of the local smallholder farmers of the area. The fresh weight of B510 inoculated plants increased significantly (approximately 1.5 times higher) in comparison with non-inoculated plants (Figure 4A). This significant effect recorded in the fresh plant weight of B510 inoculated plants was attributed to the number of panicles per plant (Figure 4B). The number of panicles per plant from inoculated rice plants exhibited a significant increase compared with non-inoculated plants. Not only did the panicles increase, but the weight of seeds, an attribute of yield improvement, showed a significant difference between inoculated plants and non-inoculated plants (Figure 4C). The grain weight recorded in inoculated plants was 1.2 times higher than in non-inoculated plants. Collectively, our results from the first field trial in 2019 suggest that the rice yield of this commercial cultivar was significantly enhanced (*p* < 0.05) by B510 inoculation; therefore, the strain B510 can potentially improve plant growth and productivity cultivars in Ghana.

In the second field trial conducted at Golinga, we examined four commercial rice varieties (AgraRice, Amankwatia, Exbaika, and Jasmine85) under a continuous irrigation system, nitrogen fertilization and B510 inoculation. The results revealed that fertilization significantly affected almost all the cultivars at the vegetative and reproductive stages (Table S2). In addition, we observed some marginal increases in the plant growth at 56 and 84 days after transplanting at the vegetative stage, which was equally significant under the chemical fertilizer treatment (+N) compared to B510 inoculation without fertilizer application (−N), among all four cultivars (Tables S2 and S3). For instance, in the +N plants, the number of tillers and the leaf chlorophyll indices recorded among cultivars were both significantly higher than the control plants (Table S3). The leaf chlorophyll content recorded during this experiment was taken as a measure of the rice cultivars’ photosynthetic response to B510 inoculation and, therefore, can be used as a direct measure of plant growth. However, the inoculation impact of B510 and the response of the rice cultivars on leaf chlorophyll index varied. In inoculated Exbaika and Jasmine85 plants, a significant increase was seen in the leafy chlorophyll index compared to non-inoculated plants 56 days after transplanting. At 84 days after transplanting, the chlorophyll content detected in inoculated Jasmine85 plants was significantly higher than inoculated Exbaika plants (Table S3).

In assessing the impact of B510 colonization on the accumulation of dry matter component in rice plants at the vegetative phase, the root length, root and shoot biomass of each treatment was measured and estimated in comparison to the control plants. This result revealed variable responses among the inoculated plants compared to +N and non-inoculated plants within the 56 and 84 days after transplanting (Table S3). Generally, the average root length recorded among all the treatments at 56 days after transplanting were approximately 4 cm longer than the length observed at 84 days after transplanting, whilst average root biomass was approximately 15 g higher at 84 days after transplanting. Root length of Amankwatia and Exbaika at 56 days after transplanting was enhanced significantly by B510 inoculation compared to their respective control plants. The average root length of AgraRice was significantly greater at 56 days in the fertilized and B510 inoculated plants (approximately 27.5 cm) compared to the control plants (approximately 19 cm). AgraRice, Amankwatia, and Exbaika root biomass were increased significantly by B510 inoculation 84 days after transplanting, compared to control plants. AgraRice and Jasmine85 shoot biomass of B510 inoculated plants at 56 and 84 days after transplanting were significantly higher than control plants. Amankwatia and Exbaika were significantly decreased by B510 inoculation after 56 days, compared to control plants. However, at 84 days, the shoot biomass of inoculated Amankwatia was significantly higher than the control plants and inoculated Exbaika plants also showed an increase in shoot biomass (although there was no detectable significant difference in the latter). These results suggest that the overall effect of B510 colonization on the growth of Ghanaian rice is positive, but the extent is variable among cultivars.

**Figure 5:**
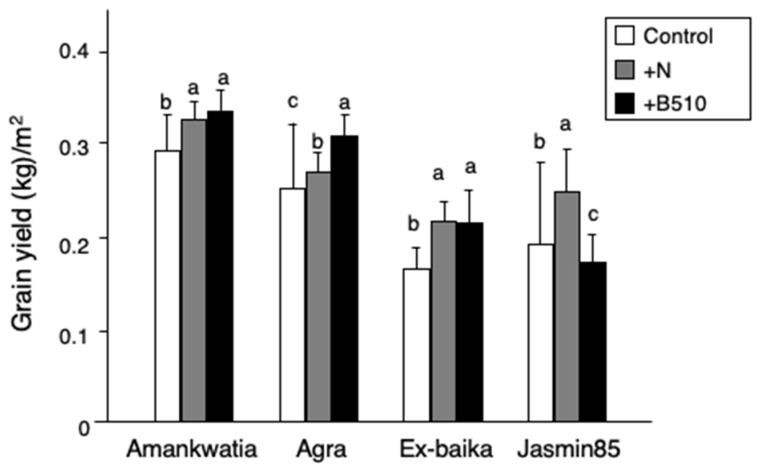
The effect of B510 inoculation on rice growth managed under field conditions in Golinga, Ghana. A field test was performed in Golinga, Ghana using 4 cultivars (Amankwatia, Agra, Ex-baika, and Jasmine85). Plants were grown in nursery field, and inoculated B510 on the young seedling. After 2 dpi, the plants were transferred to the field. Grain yield was measured after 2 months. The bar colors: non-treated control (white), applied chemical fertilizer (gray) and inoculated with B510 (black). Values presented are the average SD from three replicates per area. Statistical analysis was performed, different letters (a, b, c) indicate significant difference between treatments (Student–Newman–Keuls (SNK) test, *p* < 0.05, *n* = 9).

Other yield parameters were considered at harvest after grain maturity, including plant height, number of tillers, number of panicles, grain weight, harvest index, total biomass and grain yield (Figure 5, Table S3). Variation was found between the cultivars’ response to B510 inoculation, and the results were statistically significantly different. Inoculated AgraRice plants exhibited a highly significant increase in grain yield (kg/m^2^) compared with that of the control plants. Similarly, the grain yield of inoculated Amankwatia and Exbaika plants was significantly greater than the control plants. Jasmine85 inoculated rice plants differed in exhibiting a significant decrease in grain yield compared to the control plants.

## 4. Discussion

In this study, we firstly clarified that one of the genera of PGPR, B510 enhanced plant growth and increased the yield of some selected Ghanaian rice cultivars, including Exbaika, Ex-Boako, AgraRice, and Amankwatia. These cultivars share the following phenotypic characteristics of a short culm and high tillering capacity. Conversely, cultivars with a taller plant length and low tillering capacities like Ex-Amantin and Bawku-Market revealed a negative response to B510 inoculation (Figure 3). We can use this finding to easily identify which of the cultivars evaluated would respond positively to B510 colonization. Our results are potentially beneficial in order to reduce the risk involved in a first-time inoculation trial. Numerous *Azospirillum* species have been isolated on a worldwide basis from different plants and soils. They have been utilized as essential bio-fertilizers in crop productivity in various countries [47]. For instance, the species we used in this study were isolated from rice plants in Japan and utilized as bio-fertilizers for rice production. These bacteria are reported as non-pathogenic rhizobacteria, or endophytes, capable of promoting the growth and yield of rice plants [37,48]. Thus, they are considered to be safe as an inoculant for field evaluation.

In the present study, we confirmed the growth-promoting attributes of *Azospirillum* colonization in rice production in paddy fields under different cultivation management practices. Our results indicate that inoculation of B510, either alone or in combination with nitrogen fertilizer, significantly enhanced rice plant growth and yield-related parameters over the control treatment. Many studies have reported similar results in rice production and other crops. Ashraffuzzaman et al. [20] and Isawa et al. [37] reported that inoculation of *Azospirillum* either alone or in various combinations significantly enhanced the growth and yield of rice compared to uninoculated controls. In a recent study, Kumar et al. [49] reported that inoculation of PGPR alone or in combination significantly increased plant height up to 14% compared to the uninoculated wheat control. Furthermore, it has been shown in maize and sorghum that both PGPR alone or PGPR combined with nitrogen fertilizer increased plant dry weight compared to the control treatment [50].

The B510 strain used in this study came from a source that has been considered to enhance rice cultivars, exhibiting a short height and high tiller number phenotype. During the ‘Green Revolution’ era (1940–1960), a semidwarf1 gene was inserted into the Japanese rice cultivars from the cultivar IR-8 to reduce lodging and increase tillering capacity. Significantly, the bacteria living inside the plant tissues of these cultivars evolved to survive and become specific to their host plants, which explains the ability of B510 to enhance these cultivars. As yet, an interaction existing between *Japonica* rice cultivars and *Azospirillum* has not been reported. Although, malic acid secretion from rice roots has been reported as a contributing factor to signaling and recruiting the bacteria during symbiosis [51]. Rice roots secrete several organic acids, including malic acid, found in previous studies as a bacterial substrate, enormously triggering chemotactic responses in B510 [40]. Therefore, to investigate the interaction between Ghanaian rice cultivars and B510, we will quantify the amount of organic acids secreted during colonization and analyze the varietal responses to B510.

In our previous study, we explored the rice root microbiome from major rice-growing regions in Ghana [52]. We analyzed the 16S rRNA amplicon sequences of bacteria within the rice rhizosphere (*cv*. AgraRice and Jasmine 85) in diverse agroecological zones. We obtained 64,180 microbiome sequences (denovoOTUs) from the extracted DNA of rice roots in all sampling sites (DDBJ accession number; PRJDB9513, Table S4). Based on the 16S rRNA gene affiliation at the genus level, we identified 71 denovoOTUs belonging to *Azospirillum*. The first four denovoOTUs possessed the greatest similarity to *A. lipopherum* (denovo3368, similarity 100%), *A.* sp. TSO35-2 (denovo51236, similarity 100%), *A. agricola* (denovo1560, similarity 99.21%), and *A. brasilense* (denovo28731, similarity 99.60%). This result revealed *Azospirillum* species as the dominant strain colonized in the rice rhizosphere of Ghana. Additionally, it is worth noting that *A.* sp. TSO35-2 shared the highest similarity with the sequence from Ghana, an isolate from the rice paddy field in Tokyo, Japan [53]. The sequences of the inoculant used in this study (B510) also share high similarity to TSO35-2. It was demonstrated that *Azospirillum* is ubiquitous in soils and can inhabit plant tissues in diverse agro-ecologies across Ghana and Japan.

The application of bio-fertilizer by smallholder farmers in Ghana can increase farmers’ productivity and enhance soil fertility for sustainable production in the future. However, the demand for bio-fertilizer in Ghana is limited in comparison to developed countries. The low demand by farmers has been attributed to a lack of awareness, understanding of what bio-fertilization are and how to apply them during crop production [54]. For instance, Santos et al. [55] reported that farmers in SSA, including Ghana, use bio-fertilizers mainly as an inoculant for leguminous plants, demonstrating insufficient awareness by farmers. This contrasts with the occurrences in Argentina, South Africa, Brazil, and India, among others, where they have embraced the use of biofertilizers with promising results [56].

In general, B510 application in rice cultivation provides an insight into bio-fertilization; there are various plant responses that are important to the overall improvement of plant growth and yield by cultivars with common phenotypic characteristics, even under different agroecological conditions and farm management. The findings of this study are significant since the majority of research has focused on evaluating multiple PGPR in the first trial. Our results indicate that the utilization of B510 as bio-fertilizer could be an efficient approach in complementing chemical fertilizer requirements to sustain rice productivity, particularly in Ghana. Importantly, their application could simultaneously reduce production costs whilst restoring soil health and without harming the environment. Further research might be conducted with other release rice varieties in order to clarify the specificity mechanisms that exert beneficial effects on plant growth and development.

## Data Availability

Not applicable.

## Supplementary Materials

The following are available online at https://www.mdpi.com/article/10.3390/microorganisms9092000/s1, Figure S1: The epiphytic colonization of *Azospirillum* sp. B510 on Ghanaian rice, Table S1: Characteristics of rice (Oryza sativa L.) cultivars used in this study, Table S2: Effect of *Azospirillum* sp. B510 inoculation on plant growth promotion in Ghanaian rice (*Oryza sativa* L.) cultivars, Table S3: Effects of B510 on the reproductive stage of selected rice cultivars in field conditions under fertilizer management, Table S4: The number of *Azospirillum* OTUs detected in the rhizosphere of rice grown in Ghana.
